# FAM111B Overexpression and Immune Cell Infiltration: Implications for Ovarian Cancer Immunotherapy

**DOI:** 10.3390/biomedicines13061295

**Published:** 2025-05-24

**Authors:** Wanying Li, Fang Wei, Ting Zhou, Lijuan Feng, Lihong Zhang

**Affiliations:** Department of Oncology, Tongji Hospital, Tongji Medical College, Huazhong University of Science and Technology, Wuhan 430030, China; liwanying0115@163.com (W.L.); wfang0223@163.com (F.W.); zhouting3332006@163.com (T.Z.); fenglijuan@tjh.tjmu.edu.cn (L.F.)

**Keywords:** FAM111B, ovarian cancer, immune cell infiltration, tumor immune microenvironment, immunotherapy

## Abstract

**Background**: Ovarian cancer (OC) is characterized by high incidence and mortality rates; however, due to its immunologically “cold” phenotype, the effectiveness of immunotherapy as a strategy for OC remains inadequate. Although the FAM111B gene promotes the progression of various solid tumors, its specific function within the tumor immune microenvironment (TIME) of OC remains unclear. **Methods**: This study used multiplex immunofluorescence techniques and bioinformatics analysis to examine the role of FAM111B within the TIME of OC. Through multiplex immunofluorescence, we assessed the protein expression levels of FAM111B alongside key immune cell markers, including FOXP3, CD4, CD8, CD68, CD163, CD66b, and CD11c. Furthermore, we employed bioinformatics methods using The Cancer Genome Atlas database to validate FAM111B function at the mRNA level in OC. **Results**: We observed a positive correlation between FAM111B expression and immune cell infiltration, including T cells, macrophages, and dendritic cells. FAM111B, M2 macrophages, and regulatory T cells were associated with poorer overall survival in OC patients. Additionally, specific T cell subsets and dendritic cells were correlated positively with programmed death-ligand 1 expression, while FAM111B levels were linked to multiple immune checkpoint molecules. **Conclusions**: This study reveals a positive correlation between FAM111B overexpression and the infiltration levels of immune cells in OC. In OC patients characterized by elevated FAM111B expression, the potential augmentation of immune cell infiltration within the TIME may consequently enhance the efficacy of immunotherapy.

## 1. Introduction

Ovarian cancer (OC) is one of the most prevalent malignancies among women and is the deadliest gynecological cancer [1]. The main treatments for OC include surgery, chemotherapy, and targeted therapy [2,3].Despite these, up to 70% of patients face disease recurrence and metastasis [4], highlighting the urgent need for better treatments. While immunotherapy has been effective in various cancer [5], its success in OC is still limited. Considering the discouraging results of clinical trials focused on immune checkpoint inhibitors in OC, such as the phase II KEYNOTE-100 and NRG-GY-003 study, no immunotherapies have yet been approved for OC [6].OC is frequently classified as being immunologically “cold” due to the generation of an immunosuppressive tumorimmune microenvironment (TIME). The TIME in OC predominantly consists of immunosuppressive cells, such as regulatory T cells (Tregs) and M2 macrophages [7].The infiltration of CD8^+^ T cells and activated CD4^+^ T cells, which are integral to anti-tumor immunity, is notably limited in OC [8]. The tumor mutational burden associated with OC is also relatively low [9]. These characteristics contribute to the limited effectiveness of immunotherapy of treating OC [10]. Thus, it is crucial to better understand the TIME in OC and improve immunotherapy strategies.

Family with sequence similarity 111 member B (FAM111B) is highly expressed in multiple cancer types and is designated as a cancer nuclear protein [11,12]. It is also overexpressed in the majority of OC tumor tissue samples [11,12]. Studies suggest that FAM111B may contribute to OC progression by influencing the cell cycle, cellular migration, and apoptosis in OC cells [12]. In our prior study, we established a positive correlation between FAM111B protein expression and programmed death-ligand 1 (PD-L1) protein levels in OC tissues by immunohistochemistry (IHC) method [13]. Recent research suggests that FAM111B is crucial as a modulator of the TIME in various cancers, such as lung adenocarcinoma, renal clear cell carcinoma, head and neck squamous cell carcinoma, thymoma, and uveal melanoma [11,14,15]. However, the association between FAM111B and the TIME of OC remains poorly understood. Therefore, it is essential to undertake further studies to clarify the importance and functionality of FAM111B in the TIME of OC.

In this study, we employed multiplex immunofluorescence (mIF) analysis to examine the expression of FAM111B and the infiltration of immune cells within OC tissues. We subsequently assessed the correlation between FAM111B expression and immune cell infiltration with the prognosis of OC patients. Additionally, we conducted bioinformatics analysis using data from The Cancer Genome Atlas (TCGA) to substantiate our findings. Our findings demonstrate a positive correlation between FAM111B expression and the infiltration levels of immune cells within the TIME of OC, offering a new perspective to help identify OC patients who could benefit most from immunotherapy.

## 2. Materials and Methods

### 2.1. Tissue Microarray

This study was conducted using a tissue microarray (panelHOvaC151Su01) purchased from Shanghai Outdo Biotech Co. Ltd.(Shanghai, China), following ethical approval from the Ethics Committee of Shanghai Outdo Biotech Co. Ltd. (approval number: YBM-05-02). The tissue microarray contained tissue samples of 151 OC. The tissue samples that did not meet the required staining and imaging criteria were excluded. Among all the qualified OC tissue samples, 52.43% were diagnosed with serous carcinoma, 22.33% with mucinous carcinoma, and 25.24% with other types of ovarian tumors. Based on the International Federation of Gynecology and Obstetrics (FIGO) stage classification guidelines for OC, 31.07% of the cases were classified as TNM stage I–II, while 68.93% were classified as TNM stage III–IV. The ages of these patients ranged from 20 to 75 years, with a median age of 52 years. With respect to the histological grading of these tumors, 35.92% were classified as grade I–II, 46.60% as grade III, and 17.48% lacked histological grading information. The median overall survival (OS) of patients was 68 months.

### 2.2. Reagent

mIF analysis was performed using the PANO 7-plex IHC kit (Panovue, Cat. No.0004100100, Beijing, China). Primary antibodies utilized for the detection of protein expression included those targeting FAM111B (Novus, Cat. No.NBP1-86645, Chesterfield, MO, USA), FOXP3 (BioLegend, Cat. No.BLG 320202, San Diego, CA, USA), Pan Cytokeratin (PANCK) (Sigma, Cat. No.C2562, St. Louis, MO, USA), CD4 (ZsBio, Cat. No.ZM0418, Beijing, China), CD8 (Cell Signaling, Cat. No.CST70306, Danvers, MA, USA), CD68 (Cell Signaling, Cat. No.CST76437, Danvers, MA, USA), CD163 (Cell Signaling, Cat. No.CST93498, Danvers, MA, USA), CD66b (GeneTex, Cat. No.GTX19779, TX, USA), CD11c (Cell Signaling, Cat. No.CST45581, Danvers, MA, USA) and PD-L1 (Genetic, Cat. No.GT2280, Shanghai, China).

### 2.3. Multiplex Immunofluorescence and Immunohistochemistry

Proteins (FAM111B, FOXP3, PANCK, CD4, CD8, CD68, CD163, CD66b, CD11c) in each tissue sample were labeled via mIF. Briefly, the tissue microarray was deparaffinized by immersing it in fresh xylene for 10 min and repeating this step three times, after which it was rehydrated using an ethanol gradient (100% for 5 min, 95% for 5 min, 70% for 2 min). Heat-induced antigen retrieval was then performed. Subsequently, the sample was incubated with diluted primary antibodies specific to FAM111B (1:200), FOXP3 (1:50), CD4 (1:150), CD8 (1:200), PANCK (1:2000), CD68 (1:600), CD163 (1:300), CD66b (1:400), and CD11c (1:200) for 30 min at room temperature. Following this, the samples were incubated with secondary antibodies for 10 min at room temperature. The samples were then incubated with 100 μL of a 1× dye solution (diluted 1:100 with a signal amplification solution) at room temperature for 10 min. After staining for each marker, antigen retrieval was performed to remove antibodies, and the steps (primary and secondary antibody incubation, fluorescent staining, and antigen retrieval) were repeated for additional markers (FAM111B, FOXP3, PANCK, CD4, CD8, CD68, CD163, CD66b, CD11c). Finally, the tissue microarray was incubated with DAPI solution at room temperature. PD-L1 protein expression was evaluated using IHC methods, as previously described [13].

### 2.4. Digital Image Acquisition and Analysis

The Olympus VS200 panoramic scanning platform (Olympus, Hamburg, Germany) in conjunction with the Olympus UPLXAPO20X objective lens was employed to scan images. Subsequently, the Qupath software was used to distinguish between total, tumor, and stromal tissues, documenting the respective areas of each tissue type. Specifically, the Qupath software was utilized to construct the algorithm to precisely distinguish tumor regions (high expression of PANCK) and stromal regions (low or absent expression of PANCK). This algorithm was subsequently applied to train a classifier for segmenting the entire tissue microarray [16]. Finally, the results were reviewed and verified by the pathologists to ensure the quality of the division of tumor and stromal regions. A suitable threshold for staining intensity was established using the Qupath software. Cells exhibiting staining intensity above this threshold were classified as positive cells. The cell densities for each sample were determined by calculating the number of positively stained cells per square millimeter (mm^2^) (The description of number of positive cells for each data point is provided in Supplementary Table S1).

### 2.5. Bioinformatics Analysis

To obtain a deep understanding of the role of FAM111B within the OC TIME, we analyzed RNA sequencing data from 379 patients acquired from the TCGA database (https://portal.gdc.cancer.gov/) (accessed on 21 September 2023). We obtained transcriptomic data for genes encoding immune checkpoint proteins including PD-L1, Cytotoxic T-Lymphocyte Antigen 4 (CTLA-4), and Indoleamine 2,3-Dioxygenase 1 (IDO1), and evaluated their correlation with FAM111B expression. Additionally, we employed the XCELL algorithm to assess the correlation between FAM111B and immune score values. Subsequently, by utilizing the TIMER algorithm in conjunction with the “immunedeconv” R package, we generated heatmaps to illustrate the association between FAM111B and various immune cell types.

### 2.6. Statistical Analysis

The densities of FAM111B-positive cells and immune cell subtypes in the tissuemicroarray were measured. The normality of the data was evaluated using the D’Agostino-Pearson test. Samples were classified into groups exhibiting high and low FAM111B expression levels by the median density of FAM111B-positive cells. Differences in immune cell densities between these groups, as well as among different OC pathological types were assessed using the Mann–Whitney test. The ability of immune cell infiltration levels to predict FAM111B expression was evaluated using receiver operating characteristic (ROC) curves and the corresponding area under the curve (AUC) values (“pROC” R package). Spearman’s rank correlation analysis was used to assess the relationships between FAM111B-positive cell density, immune cell density, PD-L1 expression, and immune checkpoint expression in OC patients from the TCGA dataset. Differences in FAM111B-positive and immune cell densities between tumor and stromal tissues were analyzed using the Wilcoxon matched-pair signed-rank test. The prognostic significance of FAM111B and immune cell infiltration in OC was assessed using the Kaplan–Meier method (“ggsurvplot” R package), determining *p* values using the log-rank test. Univariate Cox regression analysis identified factors affecting OC patient prognosis. Statistical analysis was performed using GraphPad Prism (version 9.5.0), SPSS (version 27.0), or R software (version 4.0.3), with a significance threshold of *p* < 0.05.

## 3. Results

### 3.1. Multiplex Immunofluorescence Staining-Based Evaluation of FAM111B Expression and Immune Cell Subtypes in OC Tissues

**Figure 1:**
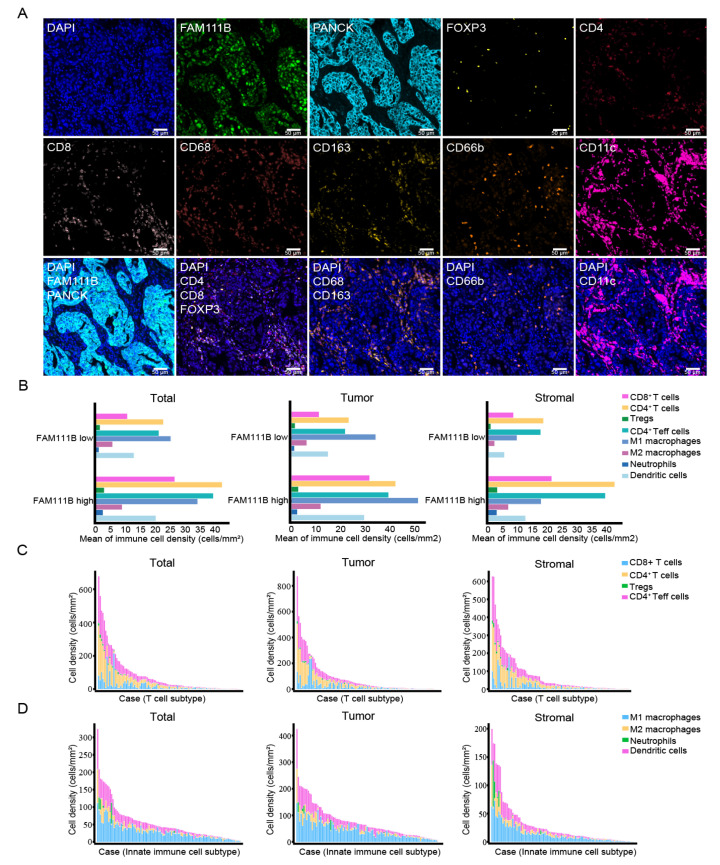
Characterization of immune cell infiltration in OC tumors expressing different levels of FAM111B. (**A**) DAPI, FAM111B, PANCK, FOXP3, CD4, CD8, CD68, CD163, CD66b, and CD11c were detected by mIF staining and are presented in blue, green, light blue, yellow, red, pink, brown, golden yellow, orange and purple, respectively. Scale bar, 50 μm. (**B**) Bar plots showing the mean density levels for different immune cell populations in total, tumor, and stromal tissues in the FAM111B high and low expression groups. (**C**,**D**) Bar plots showing the density levels of T lymphocytes (**C**) and innate immune cells (**D**) in total, tumor, and stromal tissues from each case.

To characterize FAM111B expression and immune cell subtypes present in OC tissue samples, FAM111B-positive and immune cell density levels were quantified via mIF staining. Specific cell subtypes were identified using distinct markers, each represented by a different color (Figure 1A). CD4^−^CD8^+^, CD4^+^CD8^−^, CD4^+^CD8^−^Foxp3^+^, CD4^+^CD8^−^Foxp3^−^, CD68^+^, CD163^+^, CD66b^+^, and CD11c^+^ cells, respectively, corresponded to CD8^+^ T cells, CD4^+^ T cells, Tregs, CD4^+^ effector T (CD4^+^ Teff) cells, M1 macrophages, M2 macrophages, neutrophils, and dendritic cells (DCs). PANCK served as a marker for epithelial cells. DAPI staining was employed to localize nuclei. The distributions of FAM111B-positive cells and immune cells were examined to clarify the cellular landscape within the OC TIME. The samples were categorized into groups with high and low levels of FAM111B expression based on the median density of FAM111B-positive cells (Figure 1B). Compared to the FAM111B-low group, the mean densities of different immune cell types were higher in the FAM111B-high group. Subsequently, we quantified the density of immune cells for each OC patient sample, and the density of T lymphocytes and innate immune cells are shown in Figure 1C,D.

### 3.2. FAM111B-Positive and Immune Cells Are Mainly Distributed in Tumor Tissues

**Figure 2:**
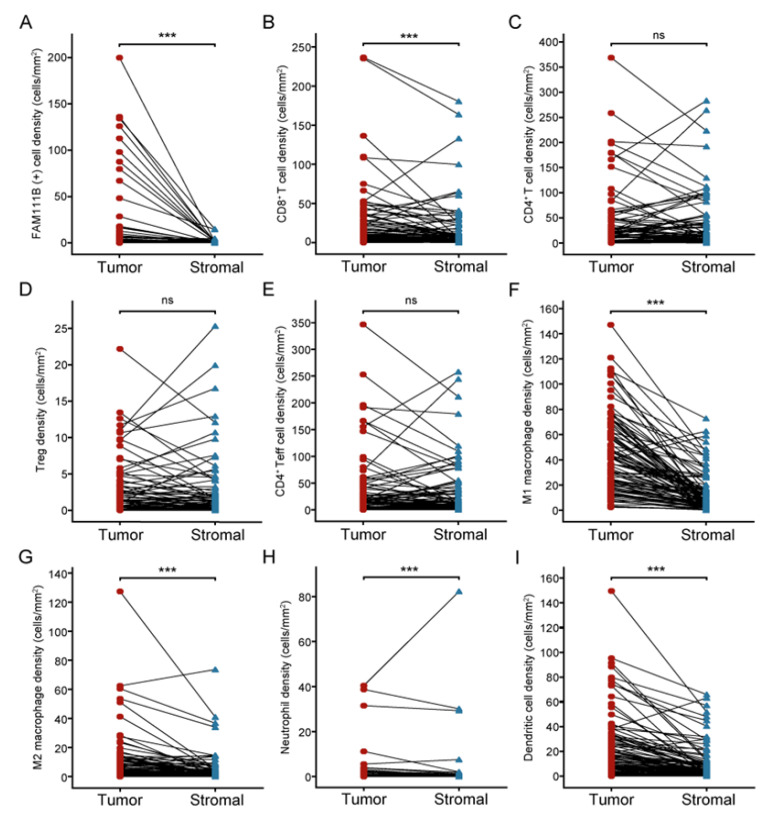
Tumor and stromal tissue samples exhibit different levels of FAM111B+ and immune cell subtypes. (**A**–**I**) Paired graphs comparing the densities of FAM111B-positive cells (**A**), CD8^+^ T cells (**B**), CD4^+^ T cells (**C**), Treg cells (**D**), CD4^+^ Teff cells (**E**), M1 macrophages (**F**), M2 macrophages (**G**), neutrophils (**H**) and DCs (**I**) between tumor and stromal tissues. Statistical analysis was performed via Wilcoxon matched-pair signed-rank test. ns, non-significant; *** *p* < 0.001.

FAM111B-positive cells were predominantly localized within the tumor tissues. Tumor tissues exhibited significantly higher CD8^+^T cell, M1 macrophage, M2 macrophage, neutrophil, and DC density levels as compared to stromal tissues (all *p* < 0.001). In contrast, the distributions of CD4^+^ T cells, CD4^+^ Teff cells, and Tregs did not differ significantly between tumor and stromal tissues (all *p* > 0.05) (Figure 2A–I). These findings indicate that FAM111B is predominantly expressed within tumors in OC patients. Additionally, CD8^+^ T cells, DCs, M1 macrophages, M2 macrophages, and neutrophils are primarily localized within OC tissues.

### 3.3. FAM111B Expression Is Positively Correlated with the Infiltration Levels of Different Immune Cells in OC Tissues

**Figure 3:**
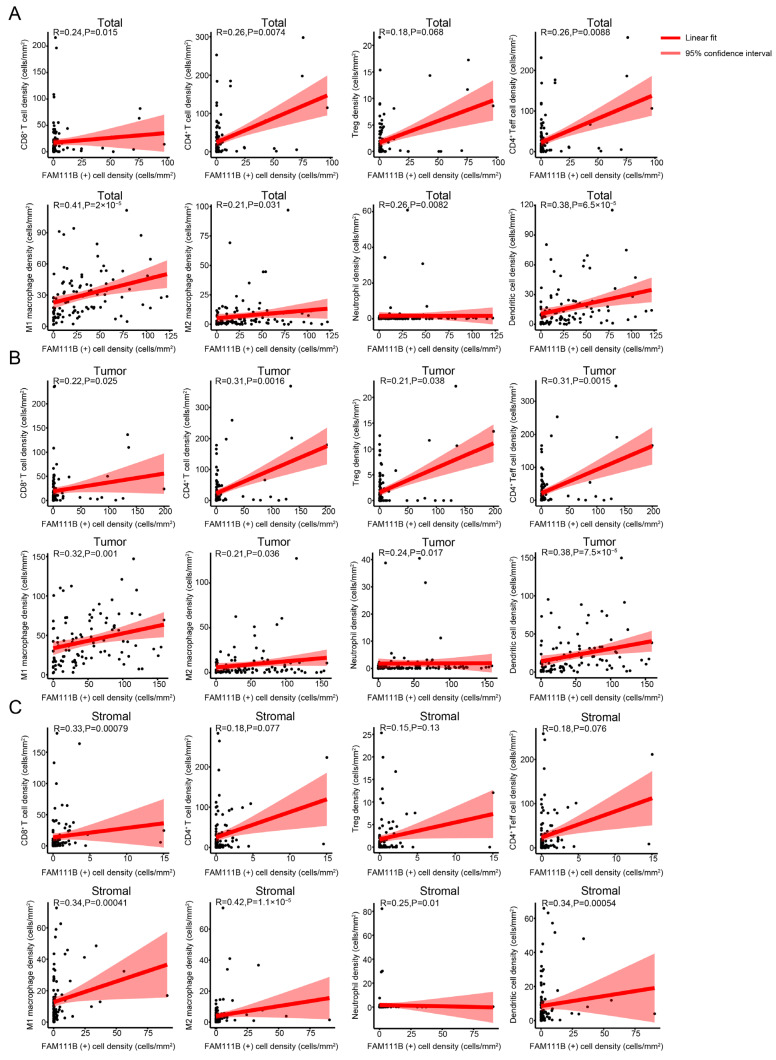
FAM111B expression is positively correlated with immune cell infiltration in OC. (**A**–**C**) Scatter plots showing the correlations between the densities of immune cells and FAM111B-positive cells in total (**A**), tumor (**B**), and stromal (**C**) tissues. Statistical analysis was performed via Spearman’s rank correlation analysis.

**Figure 4:**
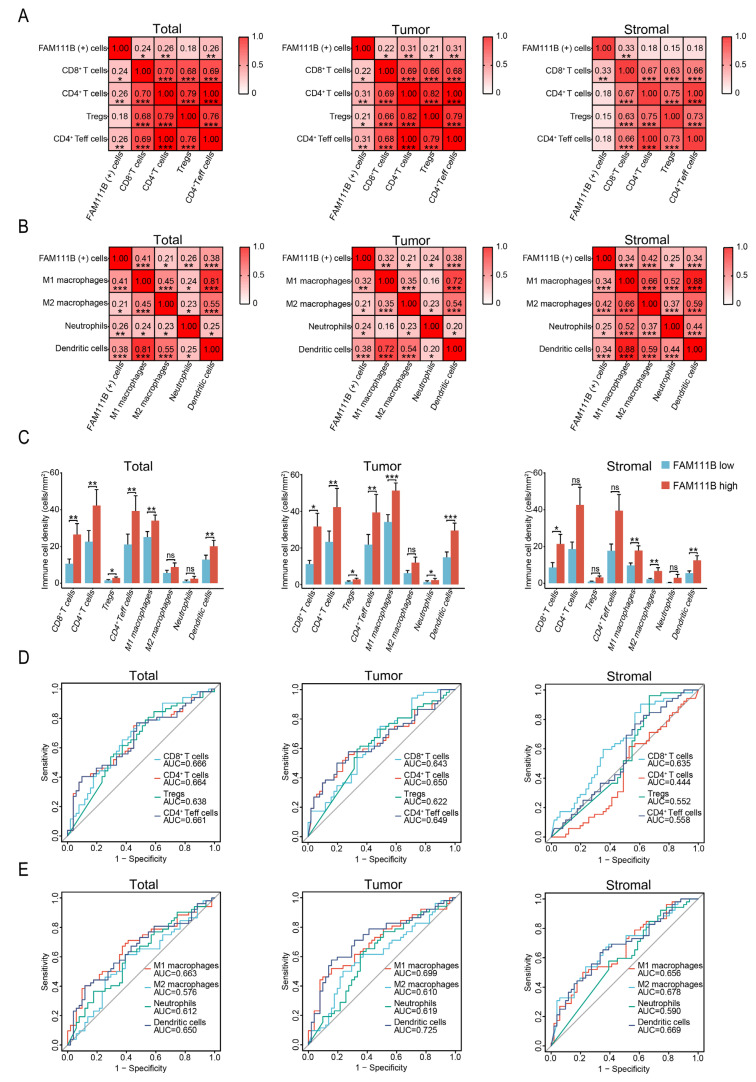
Elevated expression of FAM111B is associated with increased infiltration levels of immune cells in OC. (**A**,**B**) A matrix showing the correlation between FAM111B expression and infiltration levels of immune cells in total, tumor, and stromal tissues. Statistical analysis was performed via Spearman’s rank correlation analysis. (**C**) Bar plots comparing the densities of immune cells in total, tumor, and stromal tissues between groups with low and high levels of FAM111B expression. Immune cell density is shown as the mean ± S.E.M. Statistical analysis was performed via the Mann–Whitney test. (**D**,**E**) ROC curves corresponding to the prediction of FAM111B expression based on levels of immune cell infiltration in total, tumor, and stromal tissues. ns, non-significant; * *p* < 0.05; ** *p* < 0.01; *** *p* < 0.001.

Spearman’s rank correlation analysis was used to further investigate the association between FAM111B expression and the distribution of immune cells. Across total tissue samples, FAM111B-positive cell density levels were positively correlated with the densities of CD8^+^ T cells, CD4^+^ T cells, CD4^+^ Teff cells, M1 macrophages, M2 macrophages, neutrophils, and DCs (all *p* < 0.05). In tumor tissues, the density of FAM111B-positive cells was significantly positively correlated with the density of immune cells, including CD8^+^ T cells, CD4^+^ T cells, Tregs, CD4^+^ Teff cells, M1 macrophages, M2 macrophages, neutrophils, and DCs (all *p* < 0.05). A positive correlation was detected between FAM111B expression and the densities of CD8^+^ T cells, M1 macrophages, M2 macrophages, neutrophils, and DCs in stromal tissues (all *p* < 0.05) (Figure 3A–C and Figure 4A,B).

The relationships between FAM111B expression and the infiltration levels of different immune cell types across total, tumor, and stromal tissues were next analyzed (Table 1). In total tissues, the densities of CD8^+^ T cells, CD4^+^ T cells, Tregs, CD4^+^ Teff cells, M1 macrophages, and DCs were significantly increased in the group with high levels of FAM111B expression (Figure 4C). In tumor tissues, the group expressing high levels of FAM111B exhibited significantly higher CD8^+^ T cell, CD4^+^ T cell, Treg, CD4^+^ Teff cell, M1 macrophage, neutrophil, and DC infiltration (Figure 4C). Similarly, in stromal tissues, elevated CD8^+^ T cell, M1 macrophage, M2 macrophage, and DC densities (all *p* < 0.05) were observed in the group with high levels of FAM111B expression (Figure 4C). ROC curves further reveal that the infiltration extent for different populations of immune cells can also predict FAM111B-positive cell density, providing further confirmation of the correlation between FAM111B protein expression and the infiltration levels of immune cells in the OC TIME (Figure 4D,E).

### 3.4. Immune Cell Density Levels Are Positively Correlated with PD-L1 Protein Expression

**Figure 5:**
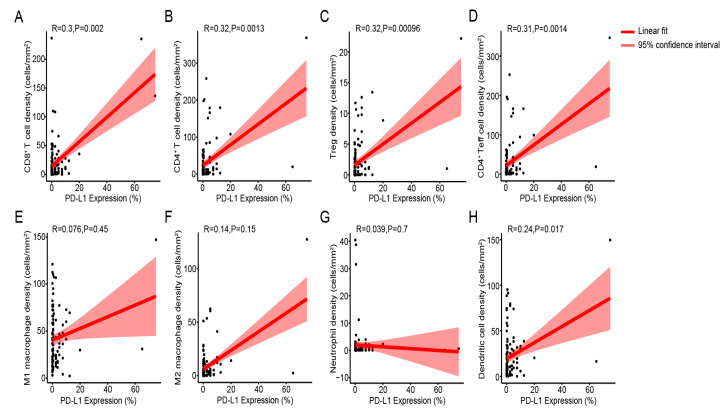
Immune cell infiltration is correlated with the expression of PD-L1 in OC. (**A**–**H**) Scatter plots showing correlations between the densities of CD8^+^ T cells (**A**), CD4^+^ T cells (**B**), Tregs (**C**), CD4^+^ Teff cells (**D**), M1 macrophages (**E**), M2 macrophages (**F**), neutrophils (**G**), and DCs (**H**) and the expression of PD-L1. Statistical analysis was performed through Spearman’s rank correlation analysis.

To assess the potential relationships between infiltration levels of immune cells and the protein-level expression of PD-L1 in tumor tissues, we conducted Spearman’s rank correlation analysis. The results of these analysis reveal a positive correlation between PD-L1 protein expression and the densities of CD8^+^ T cells (R = 0.3, *p* = 0.002), CD4^+^ T cells (R = 0.32, *p* = 0.0013), Tregs (R = 0.32, *p* = 0.00096), CD4^+^ Teff cells (R = 0.31, *p* = 0.0014), and DCs (R = 0.24, *p* = 0.017) (Figure 5A–D,H). Levels of M1 macrophage, M2 macrophage, and neutrophil density were not significantly correlated with PD-L1 expression (all *p* > 0.05) (Figure 5E–G).

### 3.5. FAM111B-Positive and Immune Cell Density Levels Are Higher in Serous Carcinoma Relative to Mucinous Carcinoma

**Figure 6:**
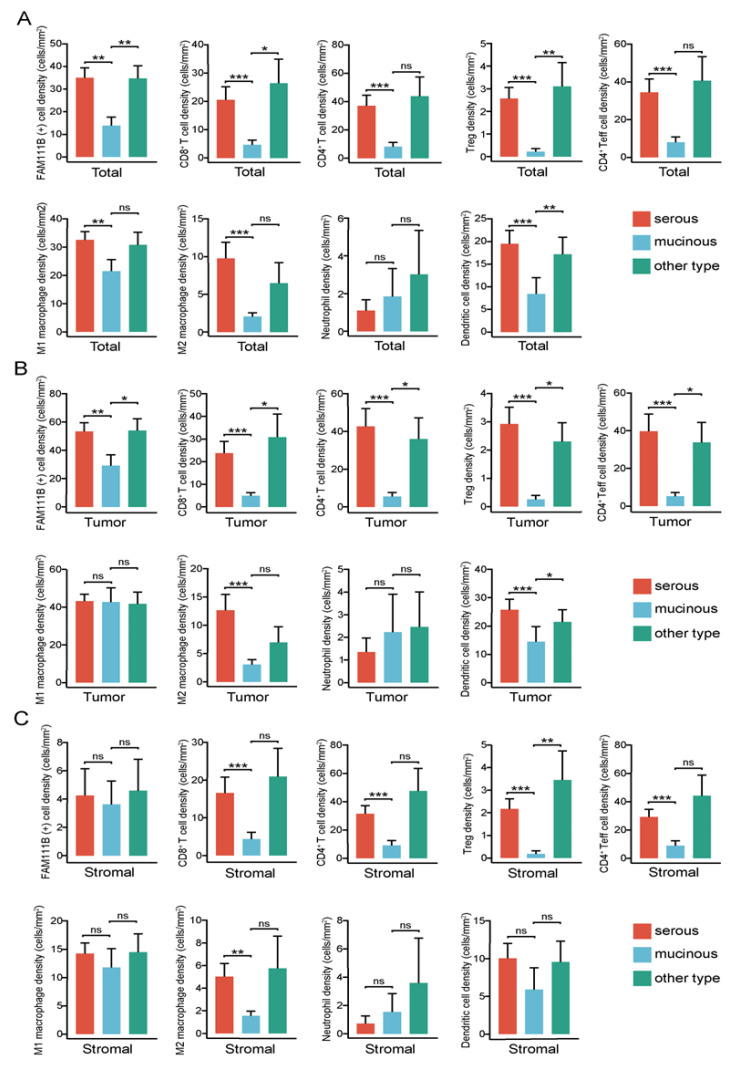
FAM111B expression and infiltration levels of immune cells are associated with OC patient pathological subtypes. (**A**–**C**) Bar plots showing a comparison of the densities of FAM111B-positive cells and immune cells between different pathological types of OC in total (**A**), tumor (**B**), and stromal tissues (**C**). All data are shown as means ± S.E.M. Statistical analysis were performed via Mann–Whitney test. ns, non-significant * *p* < 0.05; ** *p* < 0.01; *** *p* < 0.001.

In total tissues, the densities of FAM111B-positive cells (*p* = 0.0016), CD8^+^ T cells (*p* < 0.0001), CD4^+^ T cells (*p* = 0.0001), Tregs (*p* < 0.0001), CD4^+^ Teff cells (*p* = 0.0002), M1 macrophages (*p* = 0.0078), M2 macrophages (*p* < 0.0001), and DCs (*p* < 0.0001) were significantly elevated in serous carcinoma as compared to mucinous carcinoma samples. Additionally, the densities of FAM111B-positive cells (*p* = 0.0032), CD8^+^T cells (*p* = 0.0220), Tregs (*p* = 0.0041), and DCs (*p* = 0.0051) were also significantly higher in other ovarian tumor types compared to mucinous carcinoma samples (Figure 6A). In tumor tissues, FAM111B-positive cells (*p* = 0.0068), CD8^+^ T cells, CD4^+^ T cells, Tregs, CD4^+^ Teff cells (all *p* < 0.0001), M2 macrophages (*p* = 0.0002), and DCs (*p* = 0.0004) were present at higher levels in serous carcinoma samples relative to mucinous carcinoma samples. FAM111B-positive cells (*p* = 0.0178), CD8^+^ T cells (*p* = 0.0245), CD4^+^ T cells (*p* = 0.0319), Tregs (*p* = 0.0178), CD4^+^ Teff cells (*p* = 0.0367), and DCs (*p* = 0.0140) were primarily enriched in other types of ovarian tumors relative to mucinous carcinomas (Figure 6B). In stromal tissues, the densities of CD8^+^ T cells (*p* = 0.0008), CD4^+^ T cells (*p* = 0.0005), Treg cells (*p* < 0.0001), CD4^+^ Teff cells (*p* = 0.0008), and M2 macrophages (*p* = 0.0061) were significantly greater in serous carcinoma cases as compared to mucinous carcinoma cases. Additionally, the density of Tregs (*p* = 0.0051) in stromal tissues associated with other ovarian tumor subtypes was significantly higher than that observed in mucinous carcinoma cases (Figure 6C).

### 3.6. FAM111B and Immune Cell Subtypes Are Associated with the Prognosis of OC

**Figure 7:**
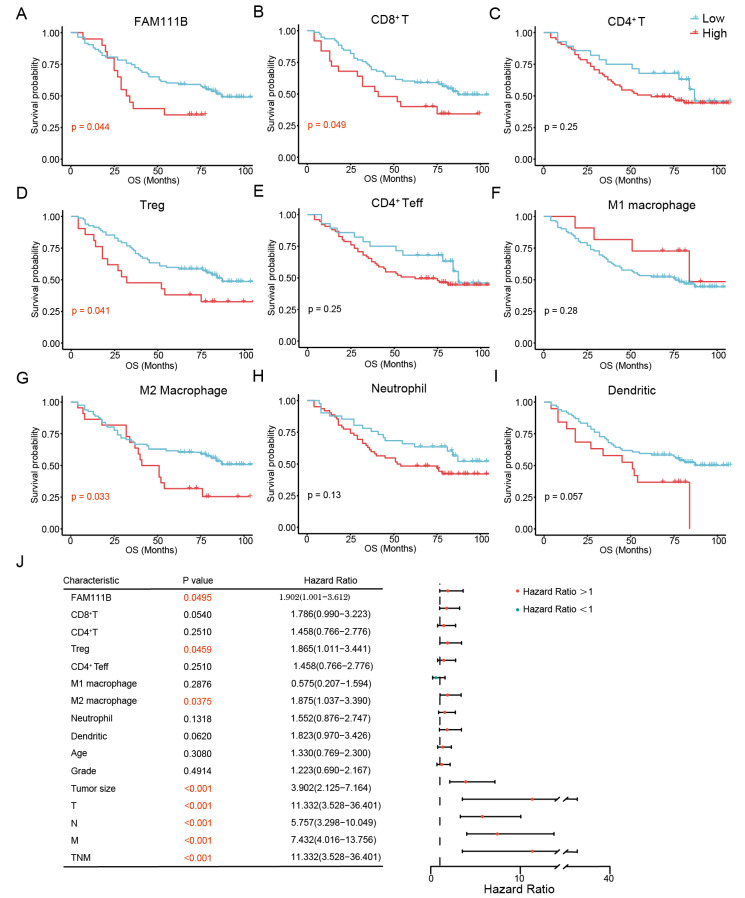
The expression of FAM111B, the infiltration levels of immune cells, and the clinical characteristics are correlated with the prognosis of OC. (**A**–**I**) Survival curves showing the relationship between OS and the densities of FAM111B-positive cells (**A**), CD8^+^ T cells (**B**), CD4^+^ T cells (**C**), Tregs (**D**), CD4^+^ Teff cells (**E**), M1 macrophages (**F**), M2 macrophages (**G**),neutrophils (**H**), and DCs (**I**) in tumor tissues fromOC patients. Statistical analysis was performed via the Kaplan–Meier method. (**J**) A forest plot showing the clinical factors and immune cell infiltration levels correlated with the prognosis of OC patients. Statistical analysis was performed via Univariate Cox regression analysis. When the *p* value was less than 0.05, it was highlighted in red.

Subsequently, we examined the relationship between FAM111B expression levels, the densities of various immune cell subtypes within tumor tissues, and OS in OC patients. Those patients exhibiting elevated FAM111B expression demonstrated significantly poorer OS (*p* = 0.044) (Figure 7A). Furthermore, higher densities of CD8^+^ T cells, M2 macrophages, and Tregs were associated with reduced OS (*p* = 0.049, *p* = 0.033, and *p* = 0.041). However, no statistically significant differences in OS were observed between groups of patients exhibiting high or low levels of CD4^+^ T cell, CD4^+^ Teff cell, M1 macrophage, neutrophil, or DC density (all *p* > 0.05) (Figure 7B–I).

Univariate Cox regression analysis indicated that elevated levels of FAM111B expression were significantly associated with reduced OS (*p* = 0.0495). Additionally, increased levels of M2 macrophage (*p* = 0.0375) and Treg (*p* = 0.0459) density were correlated with diminished OS. Tumor size, T stage, lymph node status, metastatic status, and TNM stage (all *p* < 0.001) were significantly associated with reduced OS (Figure 7J).

### 3.7. FAM111B Expression Is Associated with Immune Cell Distributions, Immune Checkpoint Expression, and Immune Scores in OC Patients from the TCGA Dataset

**Figure 8:**
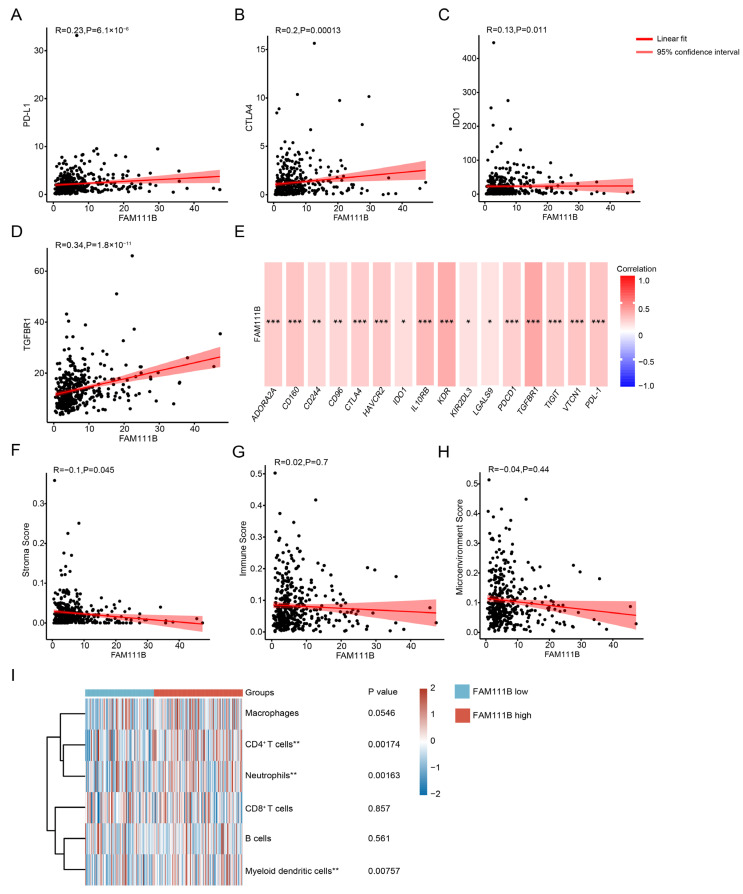
FAM111B expression is correlated with TIME characteristics in OC patients from the TCGA dataset. (**A**–**D**) Scatter plots showing the correlations between FAM111B and PD-L1 (**A**), CTLA4 (**B**), IDO1 (**C**) and TGFBR1 (**D**). (**E**) A heatmap showing the correlation between FAM111B and immune checkpoint expression. (**F**–**H**) Scatter plots showing the correlations between FAM111B and stromal score (**F**), immune score (**G**) and microenvironment score values (**H**). Statistical analysis was performed via Spearman’s rank correlation analysis. (**I**) A heatmap showing the distribution of immune cells in the FAM111B high and low expression groups. Statistical analysis was performed with the TIMER algorithm. * *p* < 0.05; ** *p* < 0.01; *** *p* < 0.001.

In this study, we used bioinformatics analysis based on data derived from the TCGA database to examine the association between FAM111B expression and the tumor microenvironment. First, we investigated the correlation between FAM111B expression and immune checkpoint molecules. This analysis demonstrated a positive correlation between FAM111B and the levels of sixteen immune checkpoint molecules (all *p* < 0.05). Notably, FAM111B expression was positively associated with key immune checkpoint molecules, including PD-L1 (R = 0.23, *p* < 0.001), CTLA4 (R = 0.2, *p* < 0.001), IDO1 (R = 0.13, *p* = 0.011), and TGFBR1 (R = 0.34, *p* < 0.001) (Figure 8A–E).

We conducted immune score analysis of samples obtained from the TCGA database. In particular, we analyzed the immune microenvironment score, which provides a comprehensive evaluation of the immune microenvironment; the stroma score, which denotes the relative abundance of stromal components within tumor tissues; and the immune score, which reflects the infiltration levels of immune cells in tumor tissues. Our findings demonstrate that FAM111B was negatively correlated with the stroma score in OC (R = −0.1; *p* = 0.045) (Figure 8F). No statistically significant correlations were identified between FAM111B expression and either the immune microenvironment score or the immune score in OC (Figure 8G,H).

Heatmap analysis revealed statistically significant variations in immune cell infiltration levels between groups with high and low FAM111B mRNA levels. Specifically, the infiltration levels of CD4^+^ T cells, neutrophils, and myeloid DCs were significantly increased in the high-FAM111B-expression group (all *p* < 0.05). However, no statistically significant differences were observed in terms of the infiltration levels of macrophages, CD8^+^ T cells, and B cells between the high- and low-FAM111B-expression groups (*p* > 0.05) (Figure 8I).

## 4. Discussion

In this study, we utilized the mIF technique to investigate the expression levels of FAM111B and the infiltration levels of immune cells within the TIME of OC. The expression of FAM111B and the infiltration of immune cells, including CD8^+^ T cells, DCs, M1 macrophages, M2 macrophages, and neutrophils, were significantly higher in OC tumor tissues compared to stromal tissues. Furthermore, our analysis demonstrated a positive correlation between the expression of FAM111B and the infiltration of various immune cell types. The infiltration levels of CD8^+^ T cells, CD4^+^ T cells, Tregs, CD4^+^ Teff cells, and DCs were positively correlated with PD-L1 expression. The presence of immune cells was significantly reduced in mucinous carcinoma samples as compared to serous carcinoma and other types of OC. Additionally, the levels of FAM111B expression, in conjunction with the infiltration levels of immune cells such as M2 macrophages and Tregs, were associated with poor prognosis in patients with OC. FAM111B expression was also positively associated with various immune checkpoint molecules. Furthermore, the results of bioinformatics analysis show that FAM111B was negatively correlated with stroma score values associated with immune suppression [17,18]. Overall, these findings indicate that FAM111B may have a significant impact on the composition of the TIME in OC.

The FAM111B protein has a trypsin-like cysteine protease domain and functions to hydrolyze proteins [19]. It plays a significant role in the development and progression of cancer. FAM111B may shape the advancement of various cancers by participating in the regulation of the cell cycle, influencing the metabolism of cancer cells, taking part in DNA damage repair, suppressing cellular apoptosis and facilitating epithelial–mesenchymal transition [20,21,22,23]. It also plays a role in the TIME of tumors. For instance, the expression of FAM111B demonstrates a positive correlation with the infiltration of multiple immune cell subtypes and the expression levels of immune checkpoint markers in lung adenocarcinoma [14,15]. Additionally, it is positively associated with the level of CD8^+^ T cell infiltration in renal clear cell carcinoma, head and neck squamous cell carcinoma, thymoma, and uveal melanoma [11]. However, to date, all extant findings concerning the role of FAM111B in the TIME are derived from bioinformatics analysis. In contrast, our study was based on the mIF detection of human tissue specimens, demonstrating that FAM111B may regulate the OC TIME.

We detected a correlation between elevated levels of FAM111B expression and the increased infiltration of CD8^+^ T cells, CD4^+^ T cells, and CD4^+^ Teff cells in both total and tumor tissues by the mIF technique. This finding aligns with previous research based on bioinformatics analysis, which indicated that FAM111B is positively associated with CD8^+^ T cell infiltration in ovarian cancer [11]. However, in stromal tissues, no significant differences were observed in the abundance of CD4^+^ T cells or CD4^+^ Teff cells when comparing groups with high and low levels of FAM111B expression. These findings support the hypothesis that FAM111B plays a role in facilitating the recruitment of a majority of T cells to tumor tissues. Thus, our results demonstrate that the analysis of the immune cell content in various areas may reflect the action of the FAM111B protein, while the underlying mechanism needs to be further explored.CD8^+^ T cells, CD4^+^ T cells, and CD4^+^ Teff cells are integral to the immune response against tumors and are positively correlated with PD-L1 expression, which is closely associated with improved responses to immunotherapy [24]. However, our analysis also revealed a significant positive correlation between FAM111B expression and the infiltration levels of Tregs, which may attenuate antitumor immune responses and promote tumor progression [25,26]. This phenomenon may be attributed to the enhanced infiltration of CD8^+^ T cells and CD4^+^ Teff cells, which secrete cytokines and subsequently recruit Tregs [27].Collectively, OC patients exhibiting high levels of FAM111B expression might benefit from immunotherapeutic interventions.

A positive correlation between the expression of FAM111B and the infiltration levels of M1 macrophages, but not M2 macrophages, was also noted in both total and tumor tissues in this study. M1 macrophages are known to mediate anti-tumor response by activating T cell activity and increasing the levels of pro-inflammatory cytokines [28]. In contrast, M2 macrophages are associated with promoting angiogenesis and facilitating tumor cell growth and metastasis [29]. Collectively, these findings suggest that FAM111B overexpression is predominantly positively correlated with the infiltration level of M1 macrophages, thereby contributing to anti-tumor immunity within total and tumor tissues. Notably, our previous study demonstrated that FAM111B expression is significantly positively correlated with the expression of ataxia telangiectasia mutated (ATM) protein, a key regulator of homologous recombination repair [30]. Mechanistically, ATM promotes macrophage polarization toward the M1 phenotype via activation of the nuclear factor-kappa B (NF-κB) signaling pathway [31,32]. These findings collectively provide a mechanistic underpinning for the positive correlation between FAM111B and M1 macrophage infiltration observed in this study.

DCs can elicit anti-tumor immunity through the presentation of tumor-associated antigens, the recruitment and activation of tumor-infiltrating T cells [33]. In this study, the expression levels of the FAM111B protein exhibited a positive correlation with the infiltration of DCs, which were positively correlated with the PD-L1 expression. This finding suggests the potential for enhanced efficacy of immune checkpoint inhibitors. [34]. Furthermore, the observed positive correlation between FAM111B levels and various immune checkpoint molecules underscores the therapeutic potential of immune checkpoint inhibitors in patients exhibiting elevated FAM111B expression. Neutrophils can be categorized into the N1 subtype, which impedes tumor progression, and the N2 subtype, which facilitates tumor progression [35]. Previous studies have shown that FAM111B can suppress the glycolytic pathway in cancer cells [22]. This reduction decreases lactate levels in the tumor microenvironment, thereby inhibiting neutrophil differentiation into the pro-tumor N2 phenotype [36].Therefore, we hypothesize that FAM111B overexpression may be associated with the infiltration level of N1 neutrophils, thereby modulating the TIME of OC. This hypothesis will be further investigated in our subsequent study.

In this study, the infiltration levels of different immune cells were markedly reduced in mucinous carcinoma samples relative to serous carcinoma and other pathological types. Recent research has further demonstrated that most mucinous carcinomas exhibit an immunosuppressive phenotype [37]. Our prior study revealed that FAM111B expression is elevated in serous carcinoma compared to mucinous carcinoma [13]. These findings imply a significant positive correlation between FAM111B expression levels and immune cell infiltration in different pathological types of OC. Therefore, we hypothesize that patients diagnosed with serous carcinoma and other pathological subtypes may experience more significant benefits from immunotherapy compared to those with mucinous carcinoma. In this study, higher levels of M2 macrophage and Treg infiltration were strongly associated with decreased OS in patients with OC. Concurrently, the increased expression of FAM111B in tumor tissues was significantly correlated with reduced OS in OC patients. These findings align with previous research [13,38,39], suggesting that FAM111B, M2 macrophages, and Tregs function as prognostic indicators of poorer survival outcomes.

Our study is subject to two primary limitations. Firstly, this analysis concentrated exclusively on ex vivo tumor tissue samples, necessitating future validation through both in vivo and in vitro studies. Secondly, while we selected key immune cell subtypes for investigation, the relatively limited number of staining markers available for mIF analysis constrained our capacity to comprehensively include all immune cell types.

## 5. Conclusions

In conclusion, our findings suggest that elevated expression of FAM111B is correlated with an increased infiltration of immune cells within the TIME of OC. The augmented infiltration levels of these immune cells may facilitate the increased efficacy of immune checkpoint inhibitors. We thus propose that analysis of FAM111B expression may aid efforts to identify OC patients who are the best candidates for immunotherapy.

## Figures and Tables

**Table 1:** Correlation between FAM111B expression and immune cell infiltration in total, tumor, and stromal tissues.

Cell Type	Total			Tumor			Stromal		
(Cells/mm^2^) (Mean)	FAM111B Low	FAM111B High	*p* Value	FAM111B Low	FAM111B High	*p* Value	FAM111B Low	FAM111B High	*p* Value
CD8^+^ T cells	0.1596–108.5405 (10.6339)	0.1825–216.0015 (26.4687)	0.0035	0–66.5236 (11.1890)	0.3183–236.5694 (31.6968)	0.0118	0–133.0868 (8.5752)	0–180.3608 (21.4257)	0.0176
CD4^+^ T cells	0.0847–252.4395 (22.6482)	0.0976–297.8265 (42.2519)	0.0040	0–178.7593 (23.3493)	0–368.7631 (42.2549)	0.0086	0–112.1557 (18.6738)	0–283.3256 (42.6883)	0.3296
Tregs	0–21.5237 (1.5045)	0–17.2489 (2.8593)	0.0141	0–12.6141 (1.5222)	0–22.1824 (2.8326)	0.0274	0–10.6848 (0.9342)	0–25.3217 (3.1866)	0.3521
CD4^+^Teff cells	0.0847–230.9158 (21.1436)	0.0976–280.5775 (39.3925)	0.0047	0–166.1453 (21.8271)	0–346.5806 (39.4222)	0.0087	0–109.7016 (17.7395)	0–258.0039 (39.5017)	0.3103
M1 macrophages	1.8225–94.1098 (25.1451)	4.6628–111.4464 (34.1032)	0.0042	2.2875–112.5114 (34.2119)	7.3032–147.1362 (51.3765)	0.0004	0–43.4280 (9.7239)	0.1643–72.6733 (17.8694)	0.0059
M2 macrophages	0–69.2255 (5.6479)	0–96.9348 (8.8280)	0.1868	0–62.4555 (6.2439)	0–127.3834 (11.9483)	0.0536	0–13.9997 (2.2146)	0–73.6824 (6.6828)	0.0017
Neutrophils	0–34.3665 (1.0833)	0–60.8612 (2.4294)	0.0509	0–38.7365 (1.2878)	0–40.4351 (2.3880)	0.0375	0–7.5640 (0.2748)	0–82.2875 (3.0029)	0.0997
Dendritic cells	0.1041–80.3256 (12.8441)	0.0356–115.0961 (20.1329)	0.0084	0–95.2228 (14.8661)	0.0661–149.5789 (29.5424)	< 0.0001	0–40.4262 (5.5252)	0–65.9148 (12.5496)	0.0028

## Data Availability

Multiplex immunofluorescence data that support the findings of this study are available from the corresponding author upon reasonable request. Data form the TCGA database was downloaded from https://portal.gdc.cancer.gov/ (accessed on 21 September 2023).

## Supplementary Materials

The following supporting information can be downloaded at: https://www.mdpi.com/article/10.3390/biomedicines13061295/s1, Table S1: The number of positive cells for each data point.

## Abbreviations

The following abbreviations are used in this manuscript:

OC	Ovarian cancer
TIME	Tumor immune microenvironment
Tregs	Regulatory T cells
FAM111B	Family with sequence similarity 111 member B
PD-L1	Programmed death-ligand 1
IHC	Immunohistochemistry
mIF	Multiplex immunofluorescence
TCGA	The cancer genome atlas
FIGO	International federation of gynecology and obstetrics
OS	Overall survival
PANCK	Pan Cytokeratin
mm^2^	Square millimeter
CTLA-4	Cytotoxic T-lymphocyte antigen 4
IDO1	Indoleamine 2,3-dioxygenase 1
ROC	Receiver operating characteristic
AUC	Area under the curve
CD4^+^ Teff cells	CD4^+^ effector T cells
DCs	Dendritic cells
ATM	Ataxia telangiectasia mutated
NF-κB	Nuclear factor-kappa B
