# Supplementary material for: FAM111B Overexpression and Immune Cell Infiltration: Implications for Ovarian Cancer Immunotherapy
Source: Biomedicines. 2025 May 24;13(6):1295. doi: 10.3390/biomedicines13061295 (PMC12189167; doi:10.3390/biomedicines13061295)
Supplement: Supplementary file 1 [file biomedicines-13-01295-s001.zip › biomedicines-3568295-supplementary.pdf]

Table S1. The number of positive cells for each data point.

Data Point	Number of positively stained cells
1	2308
2	8663
3	10169
4	7503
5	12469
6	9658
7	3005
8	8539
9	9923
10	6323
11	12185
12	6106
13	5742
14	10590
15	8225
16	5981
17	1748
18	7690
19	6639
20	2156
21	8886
22	11067
23	1774
24	9575
25	1993
26	5291
27	7589
28	5135
29	2636
30	5895
31	8147
32	5450
33	7825
34	2534
35	4113
36	5022
37	2545
38	2890
39	1394
40	3232
41	7268
42	5315

43	12098
44	2409
45	3988
46	12844
47	8796
48	3486
49	5890
50	5610
51	2921
52	1445
53	10033
54	3895
55	5509
56	3208
57	8511
58	5859
59	3494
60	6166
61	2197
62	11605
63	3961
64	8075
65	2429
66	4057
67	2921
68	5382
69	11691
70	5754
71	6650
72	12797
73	13944
74	5859
75	7598
76	9845
77	1535
78	5835
79	4641
80	9922
81	4135
82	10949
83	3372
84	11980
85	1762
86	7099

87	6321
88	8907
89	9629
90	3762
91	5089
92	14449
93	6381
94	5289
95	11157
96	11092
97	9367
98	4400
99	15943
100	3971
101	5187
102	5601
103	7045
